# RETRACTED: Arikumar et al. FL-PMI: Federated Learning-Based Person Movement Identification Through Wearable Devices in Smart Healthcare Systems. *Sensors* 2022, *22*, 1377

**DOI:** 10.3390/s26185969

**Published:** 2026-09-21

**Authors:** K. S. Arikumar, Sahaya Beni Prathiba, Mamoun Alazab, Thippa Reddy Gadekallu, Sharnil Pandya, Javed Masood Khan, Rajalakshmi Shenbaga Moorthy

**Affiliations:** 1 Department of Computer Science and Engineering, St. Joseph’s Institute of Technology, Chennai 600119, India; arikumarks@stjosephstechnology.ac.in; 2 Department of Computer Technology, Anna University, Chennai 600025, India; sahayabeni@mitindia.edu; 3 College of Engineering, IT and Environment, Charles Darwin University, Casuarina, NT 0815, Australia; mamoun.alazab@cdu.edu.au; 4 School of Information Technology and Engineering, Vellore Institute of Technology, Vellore 632014, India; 5 Symbiosis Institute of India, Symbiosis International (Deemed) University, Pune 411042, India; sharnil.pandya@sitpune.edu.in; 6 Department of Food Science and Nutrition, Faculty of Food and Agricultural Sciences, King Saud University, Riyadh 11451, Saudi Arabia; jmkhan@ksu.edu.sa; 7 Faculty of Engineering and Technology, Sri Ramachandra Institute of Higher Education and Research, Chennai 600116, India; srajiresearch@gmail.com

The journal retracts the article titled “FL-PMI: Federated Learning-Based Person Movement Identification through Wearable Devices in Smart Healthcare Systems” [1], cited above.

Following publication, concerns were brought to the attention of the Editorial Office regarding the accuracy of the authorship list and the relevance of a number of citations presented in this publication.

In accordance with the journal’s standard procedures, the Editorial Office and Editorial Board conducted an investigation. Although the authors cooperated with the investigation, the information and supporting materials provided were insufficient to verify the accuracy of the originally listed authorship, the reported author contributions, or the origin of the study. The investigation also confirmed the presence of unusual citation patterns within the article.

Consequently, the Editorial Board concluded that the concerns could not be satisfactorily resolved and has lost confidence in the integrity and reliability of this publication. As a result, the Editorial Board has decided to retract the article in accordance with MDPI’s retraction policy.

This retraction was approved by the Editor-in-Chief of the journal *Sensors*. 

The authors have been informed of this retraction and have indicated their disagreement with this decision.
